# “Dusting Off the Cobwebs”: Rethinking How We Use New Antibiotics

**DOI:** 10.3390/antibiotics14090862

**Published:** 2025-08-27

**Authors:** Jacob Myles Keck, Jacob Schultz, Alina Viteri

**Affiliations:** 1 Department of Pharmacy, University of Arkansas for Medical Sciences, Little Rock, AR 72205, USA; aviteri@uams.edu; 2 Department of Infection Prevention and Control, University of Arkansas for Medical Sciences, Little Rock, AR 72205, USA; jschultz@uams.edu

**Keywords:** antimicrobial stewardship, antimicrobial resistance, multidrug-resistant organisms, global one health

## Abstract

Antimicrobial resistance continues to escalate worldwide, threatening effective medical care, patient safety, and global health security. Traditional antibiotics are increasingly unreliable against multidrug-resistant pathogens, resulting in delayed appropriate therapy, prolonged illness, higher healthcare costs, and increased mortality. In this context, antimicrobial stewardship must evolve beyond the preservation of older drugs to include the judicious, evidence-based use of newer antibiotics. When used empirically in high-risk scenarios, novel agents can improve clinical outcomes by ensuring timely, effective coverage against MDR organisms while reducing the need for broad-spectrum combinations that drive collateral resistance and adverse effects. A major challenge, however, is the underutilization of these agents, which not only limits patient benefit but also undermines incentives for continued pharmaceutical innovation. To address this gap, stewardship programs must incorporate strategies for appropriate empiric deployment of new antibiotics, guided by local epidemiology, risk stratification, rapid diagnostics, and multidisciplinary decision-making. A coordinated global effort, linking stewardship, innovation, and policy reform, will be critical to optimize the role of novel antimicrobials in clinical practice moving forward.

## 1. Introduction

Antimicrobial resistance (AMR) represents a formidable and rapidly escalating challenge in infectious disease (ID) management, threatening the therapeutic efficacy of antimicrobial agents that have long been foundational to modern medicine. The proliferation of multidrug-resistant organisms (MDR) including extended-spectrum β-lactamase-producing *Enterobacterales* (ESBL), MDR *Pseudomonas* spp., carbapenem-resistant *Acinetobacter baumannii* (CRAB), carbapenem-resistant *Enterobacterales* (CRE), *Candida auris*, and methicillin-resistant *Staphylococcus aureus* (MRSA) has led to significant clinical and public health consequences [1,2,3,4,5,6]. These pathogens contribute to increased patient morbidity and mortality, prolonged hospitalizations, and higher healthcare costs globally [3,7,8,9,10,11]. The selective pressure exerted by inappropriate antimicrobial use, across both inpatient and outpatient settings, remains a primary driver of resistance evolution [1,4,5,6].

Antimicrobial stewardship (AMS) has emerged as an essential strategy to combat the rise in AMR by promoting the responsible use of antimicrobials [1,5]. Stewardship programs are designed to ensure that the appropriate antimicrobial is selected at the right dose and for the optimal duration (the AMS 3 D’s: correct drug, dose, and duration). This strategy optimizes clinical outcomes while minimizing adverse events and resistance selection [12,13,14]. The creation of AMS standards has been a point of emphasis for the Centers for Diseases and Prevention (CDC) over the last decade. Currently, the CDC’s core elements have become the gold standard for United States (US) hospital systems seeking guidance for AMS program initiation [15,16]. The key principles highlighted in the core elements spotlight primary interventions that have demonstrated effective antibiotic reduction in hospital systems nationwide. However, broader implementation, especially in community and low-resource settings, remains a critical focal point [5,17,18,19,20]. Additionally, the potential role of screening and judicious decolonization strategies for multidrug-resistant organisms (MDROs) is worth consideration. Nasal mupirocin or povidone iodine have demonstrated a benefit for MRSA decolonization among select patient populations, while novel approaches such as fecal microbiota transplantation (FMT) are being explored as a means to eradicate intestinal colonization with Gram-negative MDROs [21,22,23,24,25,26,27,28].

Given the global scope of AMR, antimicrobial stewardship must be integrated into international public health frameworks to ensure a sustainable, long-term impact [18,29,30,31]. The World Health Organization’s Global Action Plan on AMR underscores the role of stewardship as a pillar in the coordinated response to resistance, alongside infection prevention, surveillance, research, and investment in new therapeutics [32]. Despite growing awareness, significant disparities exist in AMS infrastructure, particularly in low- and middle-income countries where diagnostic limitations, unregulated antibiotic access, and workforce shortages pose substantial barriers [17,18,20,29,30,33]. A unified, evidence-based approach to stewardship, grounded in the principles of infectious disease epidemiology and clinical microbiology, is essential to curbing AMR and safeguarding the future of antimicrobial therapy [1,17,18,34,35,36].

The rise in MDR pathogens has exposed the limitations of older agents, many of which lack reliable activity against contemporary resistance mechanisms or are associated with significant toxicity [5,31,37,38,39,40,41]. In contrast, newer antibiotics have been specifically designed to overcome these challenges by targeting resistant organisms with improved pharmacokinetic and pharmacodynamic properties, enhanced safety profiles, and in some cases, a broader spectrum of activity [42,43,44,45,46,47,48]. These agents may allow for earlier initiation of effective empiric therapy in patients at high risk for MDR infections, reducing delays in appropriate treatment that are closely linked to excess morbidity and mortality [49,50,51]. Additionally, early initiation of appropriate therapy for MDR infections may serve as a cost-saving initiative [52,53,54]. Given the potential advantages of newer antibiotics in managing MDR pathogens, it is essential to prioritize their affordability and accessibility within clinical practice [33,46,47,55,56]. Despite their promise, utilization of these agents remains limited. Moving forward, careful attention should be directed toward preventing the phenomenon of “antibiotic trophy shelving”: a tendency to reserve novel antibiotics exclusively as last-resort options [39].

## 2. The Current Landscape of Antimicrobial Resistance

Antibiotic resistance represents one of the most pressing threats to global health, characterized by the capacity of pathogenic bacteria to evade the effects of antimicrobial agents that once reliably targeted them [1,2,4,5]. In clinical settings, the misuse of antibiotics has provided fertile ground for resistant bacteria to proliferate [1,37]. Particularly alarming is the rise in MDR organisms, such as ESBL, CRE, and CRAB [3,57]. These pathogens are increasingly responsible for healthcare-associated infections that can be difficult to treat with currently available antibiotics. Compounding the MDR problem is the use of antibiotics in agriculture [37]. This practice not only fosters resistance in zoonotic bacteria but also facilitates the dissemination of resistance genes through environmental channels. These include water runoff, soil contamination, and the food supply. Consequently, antibiotic resistance is no longer confined to hospitals or specific populations. It is a transboundary issue with widespread implications for both human and animal health [58,59,60,61,62,63,64,65].

As depicted, the ramifications of unchecked antibiotic resistance are profound. Common practices such as orthopedic surgeries, myeloablative chemotherapy protocols, and solid organ transplantation routinely rely on effective prophylactic antibiotic regimens to prevent infections [66,67,68]. As prevalence of MDR pathogens continue to rise, the risk of standard surgical prophylaxis failure will also increase (as will surgical site infections). Additionally, the economic burden of infections involving MDR pathogens is staggering, with extended hospital stays and increased drug costs (using newer/broader/more expensive antibiotics) placing significant strain on healthcare systems worldwide. Addressing this crisis requires a coordinated global response. This includes the development of novel antibiotics, stringent AMS, enhanced diagnostic tools, and public health initiatives aimed at curbing misuse and raising awareness. Without decisive action, we risk regressing to the pre-antibiotic era [69,70,71,72,73].

### 2.1. Burden of Disease from ESBL and CRE

Today, beta-lactamase enzyme producing *Enterobacterales* impact approximately 1.5 billion people globally [74,75]. Significant disease burden among majority world countries is seen alongside increasing ESBL rates in developed countries [74,75,76]. Importantly, highly studied potential sources of ESBL-producing *Enterobacterales* among human community populations includes the environment, person-to-person transmission, food, and companion animals [1,6,76]. In the US, hospitals observed ESBL-producing *E. coli* isolates increase from 7.8% in 2010 to 18.3% in 2014 [74]. These numbers have continued to rise across the US, especially for ESBL-producing pathogens isolated from urinary sources [77,78]. Additionally, Willems et al.’s meta-analysis in 2023 notated incidence of infection density for CRE (4.26 infections per 1000 patient days; 95% CI [1.69–6.82]) with cumulative incidence of infection (19%, 95% CI [15,16,17,18,19,20,21,22,23,24,25]; *p* = <0.0001; 602 cases of infection in 4547 patients colonized) at 30 days [79]. This CRE data was aggregated from sources across 26 countries, reflecting a concerning global resistome profile for MDR Gram-negative bacteria.

Both CRE- and ESBL-producing bacteria pose significant threats to patient populations due to a multifaceted set of challenges: the overuse of broad-spectrum antibiotics, difficulties in rapid identification, and inconsistent infection control practices [37]. Overuse of broad-spectrum antibiotics has been identified as a major catalyst for the emergence and spread of AMR in low- and middle-income countries [80,81,82]. Rapid detection of MDR pathogens is crucial for optimizing antibiotic choices and avoiding the overuse of carbapenems [31,83,84,85,86,87]. Furthermore, the abuse of antibiotics underscores the urgent need for rapid identification of drug-resistant bacteria at the point of care [1,74,86,88]. Without rapid identification platforms, clinicians must use patient-specific risk factors when selecting patients who might benefit from empiric broad-spectrum (carbapenem, etc.) therapy in suspected MDR infections. This underscores the nuanced clinical judgment required in antimicrobial decision-making, especially in critically ill patients (i.e., delaying antibiotics in someone with a proven infection is associated with a higher mortality rate). It also highlights the potential for unnecessary broad-spectrum antibiotic exposure when risk factors for MDR infections are not readily available and/or accessed (i.e., many critically ill patients receive broad-spectrum antibiotics regardless of risk factors) [87,89,90,91,92,93,94].

### 2.2. How Bacterial Genes Are Transferred

As has been aptly stated, emerging infectious threats can traverse borders with the ease of international travel. This underscores the urgent need for a global framework to understand and mitigate the spread of AMR. A comprehensive perspective on the molecular mechanisms underpinning resistance gene transmission is essential to guide evidence-based strategies at both local and international levels [40,95,96,97]. This imperative is driven by the alarming global rise in multidrug-resistant organisms, including those colloquially termed “superbugs,” which have been increasingly reported across diverse healthcare and community settings worldwide [1,3,4,96,98]. The primary biological processes facilitating the dissemination of antibiotic resistance determinants among bacterial populations involve horizontal gene transfer (HGT) mechanisms. These mechanisms include conjugation, the direct transfer of plasmids via cell-to-cell contact; transformation, the uptake of free DNA from the environment; transduction, the phage-mediated transfer of genetic material; and the mobilization of mobile genetic elements (i.e., integrons, transposons, and insertion sequences) [99,100,101].

### 2.3. How MDR Pathogens Colonize the Gut and Are Then Transmitted Human to Human

Recognition of the complex interplay between microbial colonization, antimicrobial resistance, and pathogenicity is essential for guiding appropriate clinical interventions [102,103]. The mere detection of MDR organisms within the gastrointestinal microbiota does not equate to active infection and, therefore, should not automatically prompt antimicrobial therapy. For instance, *Escherichia coli* is a common commensal organism in the human gut; however, only specific strains expressing virulence factors contribute to clinical disease [75,104,105,106,107,108]. From an antimicrobial stewardship perspective, the differentiation between colonization and infection is critical to avoid unnecessary antimicrobial exposure, which may further disrupt the microbiome, select for resistant phenotypes, and contribute to nosocomial transmission [102,103]. Judicious antimicrobial use must be guided by both microbiologic evidence and host-specific risk factors, grounded in evidence-based practice rather than preemptive eradication of colonizing flora. Nonetheless, documented cases of human-to-human transmission of MDR organisms, particularly in healthcare settings, necessitate a proactive but measured approach to infection prevention and control [109,110,111,112].

MDR bacteria employ multiple strategies to establish and maintain colonization within the gastrointestinal tract, thereby complicating the clinical landscape. These include mechanisms such as HGT, which facilitates the acquisition of adhesion factors that enable mucosal attachment; biofilm formation that confers environmental persistence and antibiotic tolerance; metabolic flexibility to compete for limited nutrients; evasion of host immune surveillance through modulation of innate immunity; and antagonistic interactions with surrounding microbiota that enhance competitive fitness [113,114,115,116,117,118,119]. These phenotypic traits operate synergistically to establish a resilient niche for MDR organisms. Consequently, clinical decision-making must integrate principles of antimicrobial stewardship with infection control strategies, aiming to minimize selective pressure while allowing for microbiome recovery and containment of transmission [23,112]. This underscores the importance of tailored interventions based on colonization status, clinical syndrome, and institutional epidemiology while reinforcing adherence to standard and appropriate isolation precautions when indicated.

### 2.4. Agriculture/Water and MDR Pathogens

The transmission of pathogenic microorganisms across human, animal, and environmental interfaces is governed by intricate ecological and molecular interactions [120,121,122,123,124]. Understanding the dynamics of both biotic and abiotic reservoirs, as well as zoonotic spillover of MDR organisms to human hosts, necessitates an exploration of the mechanisms underlying environmental acquisition, selection, and dissemination of resistance determinants [124,125]. Mechanisms by which environmental pressures contribute to the emergence and persistence of MDRO bacterial phenotypes include the HGT of resistance elements from natural reservoirs such as soil and aquatic ecosystems; mobilization via genetic vectors including plasmids, transposons, and bacteriophages; dissemination through contaminated surface water, blue water, and wastewater systems; selective pressure exerted by intensive agricultural practices, including the use of antimicrobials in livestock; and anthropogenic influences stemming from human antibiotic consumption and environmental contamination. Collectively, these drivers underscore the need to use a systems-level One Health approach to interrupt the global dissemination of antimicrobial resistance [58,121,122,126,127].

There are numerous pathways through which genetic material associated with antibiotic resistance (AR) is exchanged among human populations [128]. Environmental and agricultural systems play a major role in this process. For instance, the use of antibiotics in livestock feed, particularly in broiler chickens and swine, can lead to the development of resistant bacteria that are transmitted to humans through food consumption [129,130]. Additionally, agricultural runoff from land treated with pesticides, metals, and fertilizers may contribute to the selection of AR genes, even in the absence of direct antibiotic exposure [121,131]. Anthropogenic changes in soil properties—such as through contamination with manure, wastewater, and other inputs—can further influence microbial communities and resistance dynamics [92]. Urban environments also contribute through stormwater runoff and sewage systems, where human-associated microbiota and antibiotic residues enter the water cycle [121,132]. Over time, microbial communities within the built environment may shift in response to human habitation, promoting the persistence and spread of AR genes. Together, these factors highlight the interconnected nature of human, environmental, and agricultural systems in the spread of antimicrobial resistance [133,134,135,136,137].

Colonization of residents with multidrug-resistant (MDR) microorganisms in healthcare settings may be influenced by environmental factors, including shared common areas. O’Fallon et al. investigated MDR bacteria at a 600-bed long-term care facility (LTCF) in metropolitan Boston [108]. Colonization by MDR Gram-negative organisms was found to surpass that of vancomycin-resistant *Enterococcus* (VRE) and methicillin-resistant *Staphylococcus aureus* (MRSA), suggesting significant resident-to-resident transmission [138]. Shared common areas were identified as key contributors to cross-transmission, underscoring the critical role of effective infection control policies [138,139].

While environmental contamination of inanimate surfaces is a well-established factor in pathogen transmission, other pathways including foodborne exposure play important roles [140]. Animal-derived food products pose risks for foodborne bacterial infections in LTCFs, necessitating proper cooking, hygienic food handling, and surface sanitation [141,142,143]. Furthermore, person-to-person transmission, especially of viral pathogens, requires strict preventative measures such as hand hygiene, use of pasteurized food products, and regular environmental cleaning [142,143,144]. Similar infection control strategies are critical in hospital settings where patients are more vulnerable to microbial colonization and transmission [145,146,147,148,149].

### 2.5. Paradigm Shift in Approach: Infection Control and AMS in the Acute Care Environment

The molecular epidemiology of environment–animal–human transmission networks has revealed concerning impacts on the human gut resistome, including colonization by both commensal and pathogenic organisms carrying antibiotic resistance genes [122,150,151,152]. Numerous studies have demonstrated that close contact with animals and environmental sources can facilitate microbial and resistome overlap between species, contributing to the spread of antimicrobial resistance [151,153]. For example, shared environments such as farms, wastewater systems, and household settings act as reservoirs for resistant bacteria, which may transfer to humans through direct or indirect contact [121,131,152,154,155]. Infection prevention and AMS strategies must therefore evolve to incorporate concise and practical ecological frameworks emphasizing microbial community dynamics, selective pressures, and horizontal gene transfer into training programs for healthcare workers [156,157,158,159]. Importantly, the microbiome exchange between humans and the surrounding environment does not occur solely in healthcare settings—domestic animals and urban exposures also shape the human gut microbiota and resistome [152,160,161]. Urbanization and anthropogenic environmental changes have altered microbial landscapes, which in turn affect human colonization patterns and susceptibility to resistant infections [161,162,163]. As such, healthcare systems must embrace a robust and holistic approach to infection control, acknowledging the myriad environmental pressures influencing microbial ecology [137,155,164]. Without a shift in mindset and systems-level practices, global antimicrobial resistance is projected to escalate [1,3,5].

## 3. Is It Time to Remove the “New Antibiotic Trophy” from the Shelf?

The emergence and spread of MDR pathogens have necessitated the development and clinical adoption of newer antimicrobial agents with enhanced spectra of activity [165,166]. Since 2010, over 18 new antibiotics have been FDA approved. Most of these agents target MDR Gram-negative pathogens, although some agents also display extensive Gram-positive coverage. Despite this, few agents with novel mechanisms of action have reached clinical practice over the last two decades. Major decision leaders at both the hospital administration and the industry level have discussed avenues to combat the decreased output of novel antimicrobial agents, but these efforts have failed to lead to long-term solutions [41]. To find a sustainable resolution for processing and developing novel antibiotics, many factors must first be considered. We postulate there are key components contributing to the lack of production of new antibiotics. These include, but are not limited to, alterations of hospital administration landscapes secondary to COVID-19, narrow profit margins for drug manufacturers, inadequate insurance reimbursement for the treatment of MDR pathogens in the inpatient setting, drug acquisition cost, and hesitancy to utilize newer antibiotics in clinical practice. We term the latter as “antibiotic trophy shelving” [39,167,168,169,170,171,172,173,174,175,176].

In clinical practice where a high abundance of MDR pathogens are routinely seen in cultures, the use of newer agents can lead to improved patient outcomes by facilitating early, appropriate therapy. This applies particularly to high-risk populations such as immunocompromised individuals or those in intensive care units [46,177,178,179,180]. Timely initiation of effective antimicrobial treatment is a well-established determinant of clinical success and mortality reduction in severe infections—especially those involving MDR pathogens [179,180,181]. Moreover, many of the newer agents, specifically those with a beta-lactam backbone, have favorable pharmacokinetic and pharmacodynamic properties that support optimized dosing strategies. This includes extended or continuous infusions, which can enhance target attainment in critically ill patients with altered drug distribution and clearance [182,183,184,185,186]. Another possible benefit of newer antimicrobial agents lies in their role within antimicrobial stewardship frameworks. By offering targeted activity against MDR pathogens, these drugs may reduce the need for combination regimens for definitive treatment that carry a higher risk of collateral damage, including *Clostridioides difficile* infection, nephrotoxicity, and further MDR resistance development (although data does exist supporting combination usage for infections involving MDR pathogens) [184,187,188,189,190,191,192,193]. As resistance mechanisms continue to evolve, the integration of newer agents into clinical algorithms, supported by ongoing surveillance and outcomes data, will be essential to maintain the efficacy of antimicrobial therapy in an era of increasing resistance complexity [36].

## 4. How to Prevent “Antibiotic Trophy Shelving” with Newer Antibiotics

Over the past decade, many medical specialties have experienced significant progress with the introduction and adoption of novel drug therapies in clinical practice. Notably, the development and widespread use of protein-targeted chemotherapies has grown rapidly, with several becoming standard treatment options [194]. This adoption trend among clinicians fosters a positive feedback loop, where increased utilization drives further investment in research and development, largely fueled by rising profit margins [195]. However, this momentum has not extended to the field of ID, where innovation has lagged, particularly in the face of emerging MDR Gram-negative pathogens [39,196,197,198,199,200]. With AMR continuing to rise and few novel ID agents in the pipeline, there is an urgent need for targeted therapeutic advancements in this critical area [5,201].

As discussed, AMR has become a strong focal point globally and has led to the creation and implementation of AMS standards across many clinical platforms [4,5,17,18]. A common misconception of AMS practices is that they are meant to discourage clinicians from using newer, broader-spectrum, Gram-negative covering agents when their use is clinically appropriate. However, this is not the intent of most AMS programs [55]. Nonetheless, some clinicians will “shelve” newer antibiotics for fear of using them, equating to “losing” them due to resistance development [39,195]. Unfortunately, the practice of antibiotic shelving has multiple fundamental pitfalls. First, some of the recent Gram-negative antimicrobial agents approved are superior to the current standard of care (SOC) [177,178,202]. Second, newer antibiotics are often associated with favorable safety profiles compared to the SOC, especially when colistin and/or aminoglycosides are employed [203,204]. Third, delays in coverage for MDR pathogens is associated with worse clinical outcomes (i.e., delays in appropriate empiric therapy for infections involving MDR pathogens is suboptimal) [49,181]. Lastly, the availability heuristic bias secondary to growing global AMR data can lead to clinicians overestimating the true burden of resistance within their geographical region. Instead, clinicians should evaluate local resistance patterns to determine the “true” burden of AMR in their practice area [34,205,206,207]. This will prevent the clinician from falling into the clinical “trap” of providing substandard care empirically due to reservations that utilizing a new antimicrobial agent may lead to resistance. This is especially true in the setting of MDR risk factors. Instead, clinicians should be encouraged to utilize the best available therapy, especially empirically, and redirect their focus on other efforts that can slow the spread of AMR. The use of strategies such as avoiding inappropriate diagnostic testing, limiting durations of antibiotics for common infections, treating infections with targeted, guideline-driven antibiotics when MDR risk factors are not present or determined via predictive modeling, and de-escalating to the most targeted therapy when microbiological results are available is warranted [34,49,208,209].

## 5. A Case for the Use of Newer Antimicrobial Agents

The emergence of newer antimicrobial agents, such as ceftazidime–avibactam, meropenem–vaborbactam, sulbactam–durlobactam, and cefiderocol has significantly improved the management of infections caused by MDR pathogens (i.e., CRE, MDR *Pseudomonas aeruginosa*, and CRAB) [42,193,202,203,210,211,212,213,214,215,216,217]. These agents provide a therapeutic edge compared to traditional agents through expanded β-lactamase inhibition and, in some cases, novel mechanisms of cellular entry and binding [218,219]. Clinical trials and real-world data consistently demonstrate that early and appropriate use leads to better microbiological eradication, faster clinical stability, and reduced mortality, especially in critically ill patients with limited treatment options [43,44,202,220,221,222,223,224,225,226,227,228]. Their favorable pharmacokinetic/pharmacodynamic profiles allow for optimized dosing strategies, such as extended infusions, that enhance tissue penetration and efficacy in patients with altered physiology [229,230,231]. In addition, these agents reduce dependence on nephrotoxic alternatives like polymyxins and aminoglycosides, lowering the risk of adverse events [193,203]. When incorporated into antimicrobial stewardship programs, these therapies not only enhance clinical outcomes but also help mitigate resistance by preserving the microbiome and reducing reliance on legacy antibiotics [192,232,233,234,235,236,237,238]. Together, these attributes underscore the importance of incorporating newer antimicrobials into treatment protocols for severe, resistant infections. Below are brief reviews of the potential clinical scenarios where newer antimicrobial agents may offer clinical benefit over the SOC.

### 5.1. The Case for Eravacycline

Eravacycline, a novel fluorocycline within the tetracycline class, has emerged as a potent therapeutic option for the treatment of complicated intra-abdominal infections (cIAIs), particularly in the context of rising resistance to β-lactam agents [239]. Its broad-spectrum activity encompasses a range of Gram-positive, Gram-negative, anaerobic, and MDR organisms (ESBL and CRE), while notably sparing *Pseudomonas aeruginosa* [240]. In the phase 3 IGNITE-1 and IGNITE-4 trials, eravacycline demonstrated non-inferiority to ertapenem and meropenem, respectively, in terms of clinical cure rates in patients with cIAIs, underscoring its efficacy as a viable alternative to carbapenem therapy in appropriate clinical settings [241,242,243].

One key advantage of eravacycline lies in its ability to mitigate selective pressure on carbapenem agents, a critical component of antimicrobial stewardship strategies aimed at preserving last-line therapies. Unlike carbapenems, eravacycline is not susceptible to hydrolysis by carbapenemases, offering an effective option in regions with a high prevalence of CRE [240]. Furthermore, eravacycline exhibits favorable pharmacokinetic and pharmacodynamic properties, including a long half-life and high tissue penetration, which support once-daily intravenous dosing and facilitate effective concentrations at the site of infection. Lastly, eravacycline is associated with a lower incidence of *Clostridioides difficile* infection and reduced nephrotoxicity compared to broad-spectrum β-lactams, further strengthening its role as a carbapenem-sparing agent in the management of cIAIs [239,240,241,244].

### 5.2. The Case for Ceftolozane–Tazobactam

Ceftolozane–tazobactam, a combination of an antipseudomonal cephalosporin and a β-lactamase inhibitor, has demonstrated potent activity against MDR Gram-negative pathogens, most notably *Pseudomonas aeruginosa* and ESBL-producing *Enterobacterales* [42,245,246]. Additionally, this agent retains activity against MDR *Pseudomonas aeruginosa* strains housing overexpression of efflux pumps, AmpC β-lactamases, and/or loss of OprD porins [42,245,246]. Approved for the treatment of complicated urinary tract infections (cUTIs), cIAIs, and hospital-acquired/ventilator-associated bacterial pneumonia (HABP/VABP), ceftolozane–tazobactam has shown clinical efficacy in both randomized trials and real-world settings, including in patients with limited treatment options due to resistance [246,247,248,249,250,251].

One noteworthy trial for ceftolozane–tazobactam was the ASPECT-NP, which compared the agent to meropenem in adult patients with HABP and VABP. The trial demonstrated the non-inferiority of ceftolozane–tazobactam to meropenem for a 28-day all-cause mortality rate (24.0% vs. 25.3%; adjusted treatment difference −1.1%, 95% CI −8.1 to 5.9). A notable advantage emerged in a pre-specified subgroup of patients with VABP, where ceftolozane–tazobactam showed a lower mortality rate (24.2%) compared to meropenem (37.0%), suggesting a potential therapeutic benefit in this high-risk population [248]. With evidence that demonstrates activity of ceftolozane–tazobactam for meropenem-resistant *Pseudomonas aeruginosa* isolates, along with results from the ASPECT-NP trial, it is reasonable to consider the drug for empiric therapy in clinical scenarios involving critically ill patients diagnosed with VABP who have risk factors for MDR *Pseudomonas aeruginosa*.

### 5.3. The Case for Sulbactam–Durlobactam

Sulbactam–durlobactam, a novel β-lactam/β-lactamase inhibitor combination, has emerged as a promising targeted therapy for infections caused by CRAB. Sulbactam, a penicillanic acid sulfone, retains intrinsic activity against *Acinetobacter baumannii* through its binding to essential penicillin-binding proteins (PBPs), particularly PBP1 and PBP3. However, its clinical efficacy has historically been limited by degradation via class D OXA-type carbapenemases, which are prevalent in CRAB strains. The addition of durlobactam, a non-β-lactam diazabicyclooctane inhibitor, effectively restores sulbactam’s activity by potently inhibiting OXA-type β-lactamases and class A/C enzymes. The phase 3 ATTACK trial demonstrated the non-inferiority of sulbactam–durlobactam to colistin for the treatment of serious CRAB infections, with significantly improved all-cause mortality (19.0% vs. 32.3%) and a markedly better safety profile, particularly with respect to nephrotoxicity [193,203,210,252,253,254].

When compared to cefiderocol, another agent with in vitro activity against CRAB, sulbactam–durlobactam offers potential clinical advantages in both CRAB-specific targeting and resistance mitigation [254]. Although cefiderocol, an iron-chelating siderophore cephalosporin, demonstrates broad-spectrum activity, recent evidence has raised concerns regarding its efficacy in the treatment of CRAB infections, particularly following the CREDIBLE-CR trial, where a numerically higher mortality was observed in patients with *Acinetobacter* infections [193,255,256,257,258]. In contrast, sulbactam–durlobactam not only directly targets PBPs but also circumvents resistance mechanisms that limit cefiderocol’s performance, including siderophore receptor downregulation and permeability defects [193,259]. For patients with life-threatening CRAB infections, sulbactam–durlobactam represents a pathogen-focused, mechanistically rational alternative with favorable outcomes, supporting its role as a front-line agent within antimicrobial stewardship frameworks [48].

### 5.4. The Case for Meropenem–Vaborbactam

Meropenem–vaborbactam, a combination of a carbapenem and a cyclic boronic acid β-lactamase inhibitor, has demonstrated potent activity against *Klebsiella pneumoniae* carbapenemase (KPC)-producing *Enterobacterales*, which remain among the most clinically significant CRE organisms globally. Vaborbactam provides high-affinity inhibition of class A serine β-lactamases, particularly KPC enzymes, restoring meropenem’s activity against strains otherwise resistant through KPC-mediated hydrolysis [211,260]. The TANGO II trial, which compared meropenem–vaborbactam to the best available therapy (predominantly ceftazidime–avibactam), demonstrated superior clinical cure rates and reduced mortality among patients with serious CRE infections, including bloodstream infections and hospital-acquired pneumonia. Notably, meropenem–vaborbactam is associated with a lower risk of resistance emergence during therapy, a key concern with ceftazidime–avibactam, for which KPC mutations leading to treatment failure have been increasingly reported [211,213,214,260,261,262,263].

Compared to other agents that exhibit in vitro activity against the *Burkholderia cepacia* complex, a challenging pathogen particularly in cystic fibrosis and immunocompromised hosts, meropenem–vaborbactam may offer a pharmacodynamic advantage due to meropenem’s superior penetration into pulmonary and systemic tissues [212,264,265]. Given its broader stability against enzymatic degradation and favorable clinical outcomes in KPC infections, meropenem–vaborbactam is a therapeutic option for clinical practices who have either a high burden of infections involving KPC and/or have genotypic data readily available allowing for the agent’s selection based on the presences of KPC production [213].

Table 1 provides a summary of the potential clinical scenarios where new antimicrobial agents may offer an advantage for certain serious/life-threatening infections.

## 6. Limitations of Using Newer Antimicrobial Agents

Despite their promising in vitro activity and potential to combat MDR pathogens, the clinical uptake of newer antimicrobial agents remains constrained by multiple real-world limitations [39,197]. One of the foremost barriers is cost, as many of these agents, developed through prolonged and resource-intensive research pipelines, are priced at a premium compared to conventional antimicrobials [167,197]. This economic burden affects not only institutional budgets but also access in resource-limited settings, where formulary decisions are often guided by cost-containment [266,267]. Compounding these challenges is the lack of timely and reliable susceptibility testing for newer antimicrobial agents, which often leads to the unnecessary consumption of broad-spectrum antibiotics empirically [39]. Therefore, successful integration of novel antimicrobials into clinical practice requires more than regulatory approval; it demands comprehensive clinician education, enhanced diagnostic infrastructure, detailed cost-analysis studies, and robust antimicrobial stewardship programs to support the rational and effective usage of newer anti-infective agents [34,48,52,83,224,226,237,238,268].

## 7. Revitalizing Older Antibiotics for Niche Infections

The strategic repurposing of existing antimicrobial agents (defined as the use of older or previously overlooked antibiotics for new or niche clinical applications) has gained traction as a viable method for mitigating antimicrobial pressure and curbing the emergence of resistance [48,269,270,271,272,273,274,275,276]. In the context of AMS, this approach allows for the targeted treatment of specific pathogens using narrow-spectrum or underutilized agents, thereby preserving the activity of last-resort antibiotics such as carbapenems or novel β-lactam/β-lactamase inhibitor combinations. For instance, agents like fosfomycin and nitrofurantoin have demonstrated efficacy against MDR *Enterobacterales* in select clinical scenarios, such as uncomplicated urinary tract infections. By using repurposed agents when appropriate, clinicians can reduce selection pressure on the broader microbiome, slowing the propagation of resistance determinants across bacterial populations [48,270,271,273,274,276].

Repurposing antibiotics also contributes to reduced ecological disruption compared to broad-spectrum therapies, particularly when agents with favorable pharmacokinetic and pharmacodynamic profiles are used in well-defined patient populations. The careful reintroduction of agents such as minocycline for CRAB infections illustrates the potential for optimizing clinical outcomes while minimizing unnecessary exposure to newer or more potent antimicrobials [48,270,271,272,273,274,275]. When guided by local resistance data, in vitro susceptibility profiles, and stewardship protocols, the judicious use of repurposed antibiotics can not only improve therapeutic precision but also serve as a sustainable component of resistance mitigation strategies [48,270,271,272,273,274,276]. Ultimately, repurposing enhances therapeutic flexibility while reinforcing core stewardship principles aimed at extending the utility of the current antimicrobial armamentarium.

Table 2 provides a summary of the potential clinical scenarios where older antimicrobial agents may preserve the usage of newer antibiotic agents for other serious/life-threatening infections.

## 8. Conclusions

In the face of escalating AMR, an integrated approach combining rigorous infection prevention practices, robust antimicrobial stewardship, and coordinated public health strategies is essential to preserve the effectiveness of existing and emerging antimicrobial therapies. Prudent antibiotic use remains the cornerstone of resistance mitigation; clinicians must balance the imperative to minimize unnecessary exposure with the need to ensure timely and appropriate therapy. Importantly, newer antimicrobial agents, developed to overcome specific resistance mechanisms, may offer significant clinical advantages over traditional agents in select patient populations, particularly those with MDR infections [202,248]. Their thoughtful incorporation into treatment algorithms, guided by microbiologic data, pharmacodynamic principles, and local resistance patterns, represents an opportunity to optimize patient outcomes. Moving forward, advanced AMS programs must evolve to incorporate rapid diagnostics, robust cost-effective analysis, data-driven precision prescribing based on microbiological and local resistance data, and risk stratification processes to guide the deployment of newer antibiotics [52,54,224,237,268,278,279,280,281,282,283].

## Figures and Tables

**Table 1:** Potential clinical roles for newer antimicrobial agents.

Drug	Potential Clinical Indications	Clinical Pearls
Eravacycline	cIAI (VRE, CRE, ESBL, CRAB) SSTI (VRE, CRE, ESBL, CRAB, and MRSA)	Low *Clostridioides difficile* Less N/V compared to tigecycline Can be administered daily in OPAT setting Can be used as a carbapenem-sparing therapy Not renally dose-adjusted Dose adjust for Child Puge C Avoid use for bacteremia and UTI Avoid use for CRAB and *S. malt*
Ceftolozane–tazobactam	HABP/VABP (MDR *P. aerug*, ESBL *Enterobacterales*) cIAI (MDR *P. aerug*, ESBL *Enterobacterales*) cUTI (MDR *P. aerug*, ESBL *Enterobacterales*) Bacteremia (off label) (MDR *P. aerug*, ESBL *Enterobacterales*)	Potential mortality benefit compared to meropenem for HABP/VABP in ICU Does cover some streptococcus Risk of *Clostridioides difficile* Avoid underdosing: use 3 g doses for severe infection (HABP/VABP) Dose adjust for renal dysfunction
Sulbactam–durlobactam	SSTI (CRAB) HABP/VABP (CRAB)	Renal dose adjust Increase dosing frequency for augmented renal clearance Infused over 3 h Studied in combination with imipenem
Meropenem–vaborbactam	cUTI (KPC, MDR *Burkholderia* spp.) HABP/VABP (KPC, MDR *Burkholderia* spp.) cIAI (KPC, MDR *Burkholderia* spp.) Bacteremia (off label) (KPC, MDR *Burkholderia* spp.)	Renal dose adjust Increase dose for augmented renal clearance Infused over 3 h Risk of *Clostridioides difficile* Risk of seizures

cIAI: complicated intra-abdominal infections; cUTI: complicated urinary tract infection; CRAB: carbapenem-resistant *Acinetobacter baumannii*; CRE: carbapenem-resistant *Enterobacterales*; ESBL: extended-spectrum β-lactamase-producing *Enterobacterales*; g: gram; HABP: hospital-acquired bacterial pneumonia; ICU: intensive care unit; KPC: *Klebsiella pneumoniae* carbapenemase; MDR: multidrug-resistant; MRSA: methicillin-resistant *Staphylococcus aureus*; N/V: nausea and vomiting; OPAT: outpatient antibiotic therapy; *P. aerug*: *Pseudomonas aeruginosa*; *S. malt*: *Stenotrophomonas maltophilia*; SSTI: skin and skin structure infections; UTI: urinary tract infection; VABP: ventilator-associated bacterial pneumonia; VRE: vancomycin resistant *Enterococcus*.

**Table 2:** Clinical scenarios for older antimicrobial agents.

Drug	Indication (Genotypes)	Advantage over SOC
Fosfomycin	Cystitis (CRE, ESBL, VRE)	Less systemic absorption Less risk of *Clostridioides difficile* Avoid need for OPAT Favorable safety profile compared to FQ or TMP-SMX Susceptibility rates to *Escherichia coli* may be higher in specific regions compared to FQ or TMP-SMX One-time dose eliminates adherence concerns
Nitrofurantoin	Cystitis (CRE, ESBL, VRE)	Less systemic absorption Less risk of *Clostridioides difficile* Avoid need for OPAT Favorable safety profile compared to FQ or TMP-SMX Susceptibility rates to *Escherichia coli* may be higher in specific regions compared to FQ or TMP-SMX
Minocycline	Invasive infections outside the bladder (CRAB)	Less risk of *Clostridioides difficile* Highly bioavailable orally Avoid need for OPAT Less expensive vs. newer agents such as sulbactam–durlobactam or cefiderocol Better tolerated, even at higher doses, compared to other IV tetracyclines with CRAB coverage (tigecycline) MIC breakpoint for CRAB is GBP 4 but lower breakpoints may be preferred [277]
Gentamicin	Monotherapy for cystitis (ESBL, CRE)	Can be given as a one-time dose Excellent bladder penetration with high urine concentrations Less expensive vs. newer agents for CRE One-time dose often avoids known AG toxicities Susceptibility rates to *Escherichia coli* may be higher in specific regions compared to FQ or TMP-SMX One-time dose eliminates adherence concerns

AG: aminoglycosides; CRE: carbapenem-resistant *Enterobacterales*; ESBL: extended-spectrum β-lactamase-producing *Enterobacterales*; FQ: fluoroquinolones; IV: intravenous; MIC: minimal inhibitory concentration; OPAT: outpatient antibiotic therapy; SOC: standard of care; TMP-SMX: trimethoprim-sulfamethoxazole; VRE: vancomycin-resistant *Enterococcus*.

## Data Availability

No new data were created or analyzed in this study. Data sharing is not applicable to this article.
